# Efficient Regulation of CO_2_ Assimilation Enables Greater Resilience to High Temperature and Drought in Maize

**DOI:** 10.3389/fpls.2021.675546

**Published:** 2021-07-26

**Authors:** Pedro M. P. Correia, Anabela Bernardes da Silva, Margarida Vaz, Elizabete Carmo-Silva, Jorge Marques da Silva

**Affiliations:** ^1^Biosystems and Integrative Sciences Institute (BioISI), Faculdade de Ciências da Universidade de Lisboa, Lisbon, Portugal; ^2^Departamento de Biologia Vegetal, Faculdade de Ciências, Universidade de Lisboa, Lisbon, Portugal; ^3^Departamento de Biologia, Mediterranean Institute for Agriculture (MED), Environment and Development, Universidade de Évora, Évora, Portugal; ^4^Lancaster Environment Centre, Lancaster University, Lancaster, United Kingdom

**Keywords:** crop improvement, drought tolerance, food security, maize, global warming, heat tolerance, water deficit, *Zea mays*

## Abstract

Increasing temperatures and extended drought episodes are among the major constraints affecting food production. Maize has a relatively high temperature optimum for photosynthesis compared to C_3_ crops, however, the response of this important C_4_ crop to the combination of heat and drought stress is poorly understood. Here, we hypothesized that resilience to high temperature combined with water deficit (WD) would require efficient regulation of the photosynthetic traits of maize, including the C_4_–CO_2_ concentrating mechanism (CCM). Two genotypes of maize with contrasting levels of drought and heat tolerance, B73 and P0023, were acclimatized at high temperature (38°C versus 25°C) under well-watered (WW) or WD conditions. The photosynthetic performance was evaluated by gas exchange and chlorophyll *a* fluorescence, and *in vitro* activities of key enzymes for carboxylation (phosphoenolpyruvate carboxylase), decarboxylation (NADP-malic enzyme), and carbon fixation (Rubisco). Both genotypes successfully acclimatized to the high temperature, although with different mechanisms: while B73 maintained the photosynthetic rates by increasing stomatal conductance (gs), P0023 maintained gs and showed limited transpiration. When WD was experienced in combination with high temperatures, limited transpiration allowed water-savings and acted as a drought stress avoidance mechanism. The photosynthetic efficiency in P0023 was sustained by higher phosphorylated PEPC and electron transport rate (ETR) near vascular tissues, supplying chemical energy for an effective CCM. These results suggest that the key traits for drought and heat tolerance in maize are limited transpiration rate, allied with a synchronized regulation of the carbon assimilation metabolism. These findings can be exploited in future breeding efforts aimed at improving maize resilience to climate change.

## Introduction

Maize is one of the most important crops worldwide, representing 39% of the total cereal production and 27% of the total harvest area for all grain crops (FAOSTAT, 2020). According to the intergovernmental panel for climate change (IPCC), water restrictions and heat waves are among the main regional risks for food production in some of the most populated areas around the globe (Mbow et al., 2019). Without major crop improvements, each degree-Celsius increase in global mean temperature can reduce, on average, the global yield of maize by 7.2% (Zhao et al., 2017). To improve the maize yield in a changing climate, and ensure food security for the increasing world population, it is essential to comprehend how maize plants respond to fluctuations in temperature and water availability, unravel the traits that confer resilience to heat and drought stress, and develop genotypes expressing those traits.

Crop production is intimately dependent on a fine-tuned stomatal regulation since a trade-off between carbon uptake and water saving is always present. Furthermore, when subjected to high temperatures, plants usually use evaporative cooling to reduce leaf temperature, that otherwise could be detrimental to photosynthetic processes (Carmo-Silva et al., 2012; Costa et al., 2013; Lawson et al., 2018). However, in response to water shortage, higher plants close stomata to limit water losses by transpiration (Chaves et al., 2003; Nunes et al., 2009; Duque et al., 2013).

It is generally accepted that plants with C_4_ photosynthesis, as maize, evolved from C_3_ plants in response to the increase in temperature, a decrease of CO_2_ concentration in the atmosphere, and episodes of drought or salinity (Edwards et al., 2001, 2004; Sage, 2004). The revolutionary factor that allowed the success of C_4_ plants under these conditions was the anatomical and biochemical differentiation that led to the development of a CCM that increases CO_2_ concentrations at the catalytic sites of ribulose-1, 5 bisphosphate carboxylase/oxygenase (Rubisco) (Edwards et al., 2001, 2004; Sage, 2004). First, this development allowed the increase of photosynthetic efficiency by decreasing the oxygenation activity of Rubisco, and consequently photorespiration (Sage et al., 2010). Second, the increase of the optimum photosynthetic temperature, in large part by surpassing the decrease in CO_2_ solubility relative to O_2_ caused at high temperature (Sage and Kubien, 2007; Dwyer et al., 2007). Ultimately, this enables C_4_ plants to use low stomatal conductance (gs) as a water-conservative mechanism, by increasing the ratio of CO_2_ fixated per water loss through transpiration (Ghannoum et al., 2010; Osborne and Sack, 2012).

Maize uses mainly a NADP-malic enzyme (NADP-ME) type C_4_ photosynthesis, where CO_2_ is fixed in the mesophyll cells (MCs) by phosphoenolpyruvate carboxylase (PEPC, EC 4.1.1.31), leading to the formation of oxaloacetate, later reduced to malate and transported into the bundle sheath cells (BSCs). There, CO_2_ is released by NADP-ME, EC 4.1.1.31 for refixation in the Calvin–Benson–Bassham cycle (CBBC) through Rubisco, EC 2.1.1.127 (Hatch, 1987; Edwards et al., 2004).

The regulatory and catalytic properties of enzymes involved in CCM contribute to the acclimation and optimization of photosynthetic efficiency when exposed to high temperatures and drought. The activation state of C_4_-PEPC generally increases when plants are exposed to water deficit (WD) (Foyer et al., 1998; Bernardes da Silva et al., 1999; Marques da Silva and Arrabaça, 2004; Carmo-Silva et al., 2008) and the sensitivity to L-malate decreases (Carmo-Silva et al., 2008). Conversely, PEPC activity is generally not affected under high temperature (Crafts-Brandner and Salvucci, 2002; Dwyer et al., 2007) and the sensitivity to L-malate slightly increases (Crafts-Brandner and Salvucci, 2002). Usually, C_4_-NADP-ME activity decreases or is not affected under WD (Du et al., 1996; Marques da Silva and Arrabaça, 2004; Carmo-Silva et al., 2008). The activation state of Rubisco was reported to mainly decrease at high temperature, in isolation, or in combination with WD (Crafts-Brandner and Salvucci, 2002; Salvucci and Crafts-Brandner, 2004a; Perdomo et al., 2017). The decline is mainly due to the thermal sensitivity catalytic chaperone of Rubisco, Rubisco activase (Rca), that otherwise promotes the release of inhibitory sugar phosphates from active sites of Rubisco (Portis, 2003; Salvucci and Crafts-Brandner, 2004a; Carmo-Silva and Salvucci, 2011; Carmo-Silva et al., 2015).

Differentiation between MC and BSC localization and functions also altered their specific energy requirement. The MCs are characterized by high grana-containing chloroplasts with higher photosystem II (PSII) activities and linear electron flow, producing ATP and most of the reducing power (NADPH). The BSCs primarily produce the ATP required in CBBC, via cyclic electron flow around photosystem I (PSI), and grana stacks are rare in these chloroplasts (Woo et al., 1970; Walker and Izawa, 1979; von Caemmerer and Furbank, 2016). Environmental regulation of the PSII levels in the BSCs chloroplasts has not been extensively examined (Meierhoff and Westhoff, 1993; Omoto et al., 2009). However, balancing the energetic capacity of the two compartments might be a requirement for the plasticity of the decarboxylating process under varying environmental conditions (Chapman and Hatch, 1981; Furbank, 2011; von Caemmerer and Furbank, 2016; Arrivault et al., 2017). The effects of high temperature and WD on the electron transport capacity can also limit the photosynthetic efficiency mainly by the impairment of the physical integrity of electron transport components of the photosynthetic apparatus (Salvucci and Crafts-Brandner, 2004a).

Previous studies focused on C_4_ photosynthesis under isolated or rapidly imposed stresses. Thus, there is scarce information about the regulation of CCM in acclimatized plants under high temperature in isolation or combination with WD. Moreover, as great diversity is observed between C_4_ metabolic types (Carmo-Silva et al., 2008; Furbank, 2011; Rao and Dixon, 2016), and even between genotypes of the same species, depending on breeding history and genome-environmental interactions (Lopes et al., 2011; Khoshravesh et al., 2020), it is crucial to study the response of genotypes with contrasting behavior.

In this study, the contrasting levels of tolerance to drought and heat of two maize genotypes, B73 and P0023, were explored to (1) characterize the combined effects of high temperature and WD on maize photosynthesis, (2) test the hypothesis that CCM efficiency is a major player in the tolerance to these conditions, and (3) ultimately unravel mechanisms that can help the improvement of maize growth under high temperatures and extended drought. The B73 genotype is heat and drought-sensitive (Petolino et al., 1990; Chen et al., 2012; Yang et al., 2018), the most widely used inbred line in breeding programs (Duvick et al., 2010; Chen et al., 2012) and one of the first maize genotypes to be sequenced (Schnable et al., 2009; Jiao et al., 2017). The P0023 genotype is a drought-tolerant Pioneer Optimum AQUAmax^TM^ hybrid line (DuPont Pioneer). The AQUAmax hybrids were developed in a long-term improvement program to increase maize yield for the US corn-belt, by applying multi-environment phenotyping and molecular markers-based selection (Cooper et al., 2014; Gaffney et al., 2015). The effects of long-term growth WD (WD25), high temperature (well-watered plants at 38°C, WW38), and their combination (WD38) on the photosynthetic efficiency was assessed by steady-state gas exchange and chlorophyll *a* fluorescence and related to (1) biomass production, (2) leaf evaporative cooling system and water balance, (3) the catalytic activity of key enzymes involved in maize CCM, (4) kinetics of photosynthetic electron transport, and (5) leaf tissue spatial heterogeneity of PSII activity.

## Materials and Methods

### Plant Material and Growth Conditions

Based on the evidence of contrasting drought and heat tolerance in previous studies (Chen et al., 2012; Cooper et al., 2014; Gaffney et al., 2015; Yang et al., 2018), two *Zea mays* L. (maize) genotypes were selected: B73, a heat and drought-sensitive line, and P0023, a drought-tolerant Pioneer Optimum AQUAmax^TM^ hybrid line (DuPont Pioneer). Plants of both genotypes were grown from seeds in a controlled environment chamber (Fitoclima 5000 EH, Aralab) in 2 L pots containing horticultural substrate (Compo Sana Universal, Compo Sana). The light was provided by fluorescent lamps (Osram Lumilux L 58W/840 cool white lamps) placed at specific distances from the plants to obtain an average photosynthetic photon flux density (PPFD) of ~300 μmol m^–2^ s^–1^ at the top of the canopy, with a photoperiod of 16 h. All plants were initially grown under a control temperature (25/18°C day/night), with 50% relative humidity (RH) for 21 days (21 days after sowing, DAS). For experiments under control temperature, plants remained at 25/18°C (day/night) with 50% RH throughout the experiment. Following 21 DAS, plants were randomly assigned to two irrigation treatments: five plants per cultivar were maintained well-watered (WW; minimum 80% field capacity, WW25) throughout the experiment and five plants were subjected to WD (WD25) for 7 days. For experiments under elevated temperature, the 21 DAS plants were also exposed to high temperatures (38/31°C day/night) with 60% RH and were randomly assigned to the irrigation treatments: five plants per cultivar were maintained WW (80±5% field capacity, WW38) and five plants were subjected to WD (WD38), for 5 days. Water deficit was established by withholding watering and sustaining a minimum of 30±5% field capacity. The water stress period was reduced to 5 days under high temperature to compensate for the higher evapotranspiration demand. The soil water content was determined gravimetrically by weighing the pots and irrigation was provided to compensate for evapotranspiration and maintain field capacity in the WW and WD pots. Leaf samples collection for biochemical analyses occurred at the end of the respective temperature and irrigation treatment, 5–7 h after the start of the photoperiod. Samples were rapidly frozen into liquid nitrogen and stored at −80°C.

### Leaf Water Status

The plant water status was estimated by leaf water content (LWC) following the methodology described by Gameiro et al. (2016). A fresh leaf sample (3–4 cm^2^) was collected at the same time as the leaf samples collected for biochemical analyses, the leaf fresh weight (LFW) was immediately measured on an electronic scale (Sartorius BP221S) and the leaf dry weight (LDW) was measured after oven drying samples at 70°C for 48 h, and LWC calculated as follows:

$$\text{LWC}=\frac{\left(\mathrm{LFW}-\mathrm{LDW}\right)}{\left(\mathrm{LFW}\right)}.$$

The leaf water potential (LWP) was measured with a C-52 thermocouple chamber (Wescor), 20 mm^2^ leaf disks were cut and equilibrated for 30 min in the chamber before the readings were recorded by a water potential datalogger (PSYPRO, Wescor) operating in the psychrometric mode.

### Above-Ground Biomass and Growth Rate

At the end of the experiment, plants were harvested to measure above-ground biomass in the form of fresh weight (FW) and dry weight (DW). The FW was directly measured in an electronic scale (Sartorius BP221S) and the DW measured after oven drying samples at 70°C for 52 h. Additionally, to assess the relative growth rate (RGR) during the acclimatization period, the above-ground biomass was measured in a random group of plants before stress imposition (21 DAS, *n* = 5). The RGR for each plant, based on the DW, was estimated as:

$$\text{RGR}=\text{ln}\frac{\left(\mathrm{W2}/\mathrm{W1}\right)}{\left(\mathrm{T2}-\mathrm{T1}\right)},$$

where W1 and W2 are the DW of each plant at time points T1 (21 DAS) and T2 at the end of the acclimatization period.

### Thermal Imaging

Thermal images were obtained using a thermal camera (Flir 50bx, FLIR Systems Inc.) with emissivity set at 0.95 and at, approximately, 1 m distance from the plants. Prior to each set of measurements, the background temperature was determined by measuring the temperature of a crumpled sheet of aluminum foil in a similar position to the leaves of interest with the emissivity set at 1.0 following the methodology described by Costa et al. (2013). Thermal images were analyzed with the software FLIR Tools (FLIR Systems, Inc.). The temperature of each plant was determined from the temperature of five leaves using the function *area*.

### Steady-State Gas Exchange and Chlorophyll *a* Fluorescence

Parallel measurements of photosynthetic gas exchange and chlorophyll *a* fluorescence were performed in a non-detached fully expanded leaf from each plant with a portable fluorescence and gas exchange system (Li-6400-40, Li-Cor Inc.), in the climatic growth chamber. The control air temperature was set to the growth temperature, 25°C (WW25 and WD25) or 38°C (WW38 and WD38), PPFD at the leaf level (I) set to 600 μmol m^–2^ s^–1^ and the CO_2_ concentration in the leaf chamber set to 400 μmol CO_2_ mol^–1^, allowing the leaf to reach steady-state assimilation rate (A) and stomatal conductance (gs). All the photosynthetic parameters were calculated by the Li-6400-40 software. A, gs, and transpiration rate (E) were calculated according to von Caemmerer and Farquhar (1981). The PSII effective quantum yield (*Φ*PSII) was obtained according to Genty et al. (1989) and electron transport rate (ETR) was then calculated as:

ETR=0.5ΦPSII×PPFD×0.84.

### Chlorophyll *a* Fluorescence Induction

The kinetics of the rapid rise in fluorescence induction was recorded on fully expanded dark-adapted leaves (10 min) exposed to a saturating light pulse (3,500 μmol m^–2^ s^–1^) for 1 s, in order to obtain the OJIP Chl *a* fluorescence transient rise (Handy PEA, Hansatech Instruments). The fluorescence parameters derived from the extracted data, namely specific energy fluxes per QA-reducing PSII reaction center and photosynthetic performance indexes were calculated according to Strasser and collaborators (Strasser et al., 2004; Tsimilli-Michael and Strasser, 2008) with the nomenclature presented in Stirbet and Govindjee (2011).

### Chlorophyll *a* Fluorescence Imaging and Rapid Light Curves

Chlorophyll *a* fluorescence rapid light curves (RLCs) were measured with a chlorophyll fluorescence imaging system (Imaging-PAM M-series Mini version, Heinz Walz GmbH) and images analyzed using the Imaging Win analytical software (Heinz Walz GmbH). The measurements were recorded in full dark-adapted leaves (10 min) with an eight-step protocol (0, 43, 111, 223, 402, 624, 782, 996 μmol m^–2^ s^–1^) with light irradiance increasing at 90 s intervals. The ETR values were extracted from representative areas of interest (AOI) for the total leaf area (L ETR), mid-veins (MV ETR), and regions between mid-veins (BMV ETR). The data were fitted to a three-parameter photosynthesis-irradiance model (Platt et al., 1982) using a derivative-free optimization by the quadratic approximation algorithm (bobyqa) in the R package phytotools (Silsbe and Malkin, 2015). The maximum ETR (ETRmax) was estimated by the following equation:

$$\text{ETRmax}={\text{P}}_{\text{s}}\left[\alpha /\left(\alpha +\beta \right)\right]{\left[\beta /\left(\alpha +\beta \right)\right]}^{\beta /\alpha },$$

where Ps is a scaling factor defined as the maximum potential ETR, α the initial slope of the RLC before the onset of saturation and β the slope of the downturn of the curve characterized by photoinhibition. As leaf ETR (L ETR) was extracted in an AOI exclusively containing MVs and BMVs, the contribution of MV and BMV ETR to the L ETR was calculated by normalizing the data to L ETR under WW25 and expressed as a relative percentage (% of WW25 L ERT).

### Enzymes Extraction

Soluble enzymes were extracted by grinding frozen leaf samples (0.1–0.3 g FW) in a cold mortar with quartz sand, 1% (w/v) insoluble polyvinylpyrrolidone (PVP), ice-cold extraction medium (1/10 FW per mL) containing 50 mM Bicine–KOH pH 8.0, 1 mM ethylenediaminetetraacetic acid (EDTA), 5% (w/v) PVP25000, 6% (w/v) polyethylene glycol (PEG_4000_), 10 mM 1,4-dithiothreitol (DTT), 50 mM β-mercaptoethanol, and 1% (v/v) protease inhibitor cocktail for plant extracts (Sigma-Aldrich), as described in Carmo-Silva et al. (2008). The leaf extracts were then centrifuged at 14,000 *g* and 4°C for 3 min, supernatants were used for enzymatic activity assays and maintained on ice until all measurements were completed. The total soluble protein (TSP) content was quantified according to the Bradford method (Bradford, 1976) using BSA Fraction V as standard protein.

### Phosphoenolpyruvate Carboxylase Activity and Sensitivity to Malate Inhibition

The PEPC activity was measured by coupling the carboxylase reaction with malate dehydrogenase (MDH) NADH-dependent, as described by Carmo-Silva et al. (2008). The maximal activity (Vmax), was determined under optimal pH (pH 8.0), saturating substrates and cofactor conditions, and 5–20 μL of leaf extract. The physiological activity (Vphysiol) was determined under similar conditions but at sub-optimal pH (pH 7.3) and PEP concentration (2.5 mM PEP). The reaction mixtures, with all the components except NADH and extract, were allowed to equilibrate at 25 or 38°C; then the enzyme extract was added, and the reaction started by the addition of NADH. The activation state of PEPC was calculated as:

$$\mathrm{Activation}\mathrm{state}=\frac{\text{Vphysiol}}{\text{Vmax}}\text{×}100.$$

For the determination of PEPC sensitivity to L-malate inhibition, a sub-sample of protein extract was desalted by gel filtration (Sephadex G-25, Sigma-Aldrich) and assayed in the same conditions as described for the Vphysiol, with 40–60 μL of the desalted extract and by measuring the activity in the absence and increasing concentrations of L-malate. The malate sensitivity was estimated by the calculation of half-maximal inhibitory concentration (IC50) and used to evaluate PEPC phosphorylation state. Reactions were measured in continuous assays by monitoring absorbance at 340 nm and carried out in triplicates.

### NADP-Malic Enzyme Activity

The NADP-ME activity was determined, according to Carmo-Silva et al. (2008). The maximal activity (Vmax), was determined under optimal pH (pH 8.0), saturating substrates and cofactor conditions, and 10–20 μL of leaf extract. The physiological activity (Vphysiol) was determined under similar conditions but with the leaf extract L-malate endogenous concentration. The reaction mixture, with all the components except NADP^+^ (Sigma-Aldrich), L-malate, and extract, was allowed to equilibrate at 25 and 38°C. Then the protein extract and NADP^+^ was added and the reaction started by the addition of L-malate (Vmax) or by the endogenous L-malate (Vphysiol). The activation state of NADP-ME was calculated as the ratio of Vphysiol to Vmax, as previously presented for PEPC. The reactions were measured in continuous assays by monitoring absorbance at 340 nm and carried out in triplicates.

### Rubisco Activity

The rubisco activities were measured by the incorporation of ^14^CO_2_ into acid-stable products at 25 and 38°C, following the protocol described in Parry et al. (1997) with modifications. The reaction mixture contained 100 mM Bicine–NaOH pH 8.2, 40 mM MgCl_2_, 10 mM NaH^14^CO_3_ (7.4 kBq μmol^–1^), and 0.4 mM ribulose 1,5-bisphosphate (RuBP, Sigma-Aldrich). The Rubisco initial activity (Vi) was determined by adding the supernatant to the mixture and stopping the reaction after 60 s with 10 M HCOOH. Total activity (Vt) was measured after incubating the same volume of extract for 1 min with all the reaction mixture components except RuBP in order to allow carbamylation of all the Rubisco available catalytic sites. The reaction was then started by adding RuBP and stopped as above. All measurements were carried out in triplicate, and control reactions were quenched with HCOOH prior to the addition of RuBP. The mixtures were completely dried at 70°C overnight and the residues re-hydrated in 0.5 mL ddH_2_O, then mixed with 5 mL scintillation cocktail (Ultima Gold, Perkin-Elmer). Radioactivity due to ^14^C incorporation in the acid-stable products was measured by liquid scintillation counting (LS7800, Beckman). The activation state of Rubisco was calculated as the ratio,

$$\mathrm{Activation}\mathrm{state}\mathrm{Rubisco}=\frac{\text{Vi}}{\text{Vt}}\text{×}100.$$

All measurements were carried out in triplicate.

### Statistical Analysis

The statistical significance of trait variation was tested by factorial ANOVA, and *post hoc* comparison between treatments was performed with Duncan test (*P* < 0.05) using IBM SPSS Statistics, Version 25 (IBM, United States). The *t*-test (*P* < 0.05) was used to analyze significant differences between experimental temperatures in the same genotypes and condition. To link the activity of enzymes involved in CCM with photosynthetic performance, a regularized canonical correlation analysis (rCCA, González et al., 2008) was employed and Clustered Image Maps (CIM or heatmaps) computed, as implemented in González et al. (2012). The analysis was performed using the mixOmics R package v 6.10.9 (Rohart et al., 2017). In this approach, a pair-wise similarity matrix is computed as the correlation between the two types of projected variables onto space spanned by the three first components retained in the analysis, and the values in the similarity matrix can be seen as a robust approximation of the Pearson correlation (González et al., 2008).

## Results

### Effects of Drought and High Temperature on Maize Photosynthetic Efficiency

**FIGURE 1:**
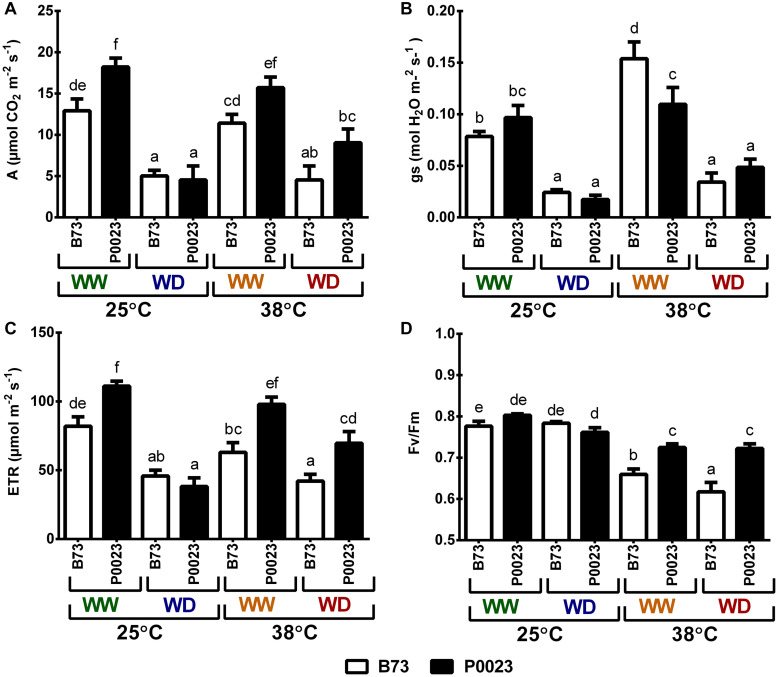
Steady-state photosynthesis in two maize genotypes (B73, P0023) grown under WW and WD conditions and acclimatized to 25 or 38°C. **(A)** Net CO_2_ assimilation, **(B)** stomatal conductance, **(C)** ETR and **(D)** maximum quantum yield of primary PSII photochemistry (Fv/Fm) were measured at 600 μmol m^–2^ s^–1^ and 400 μmol CO_2_ mol^–1^ in fully expanded leaves from 4-week-old maize plants. Values are means ± SEM (*n* = 5 biological replicates). Different letters denote statistically significant differences between treatments (Duncan analysis, *p* < 0.05).

Steady-state photosynthetic gas exchanges and chlorophyll *a* fluorescence were assessed to quantify the photosynthetic performance of B73 and P0023 under high temperature and drought. The WD plants had significantly lower net photosynthesis assimilation rate (A), stomatal conductance (gs), and ETR compared to the WW plants (Figures 1A–C). However, under WW, the genotypes modulated gs/A differently in response to high temperature. The B73 genotype increased gs to maintain A at similar levels as control (WW25) when the temperature increased and P0023 could achieve similar assimilation, as control, maintaining the same gs (Figures 1A,B). Under WD38, P0023 demonstrated a slightly higher A and significantly greater ETR than B73 (Figures 1B,C). The maximal quantum efficiency of PSII (Fv/Fm) was highly affected by the increase of temperature in B73 (Figure 1D). In this genotype, the decrease in FV/Fm with temperature was more pronounced under WD (WD38), whereas in P0023, the Fv/Fm decreased similarly in both irrigation regimes at 38°C (Figure 1D).

### Leaf Water Status and Biomass Allocation Under Drought and High Temperatures

**FIGURE 2:**
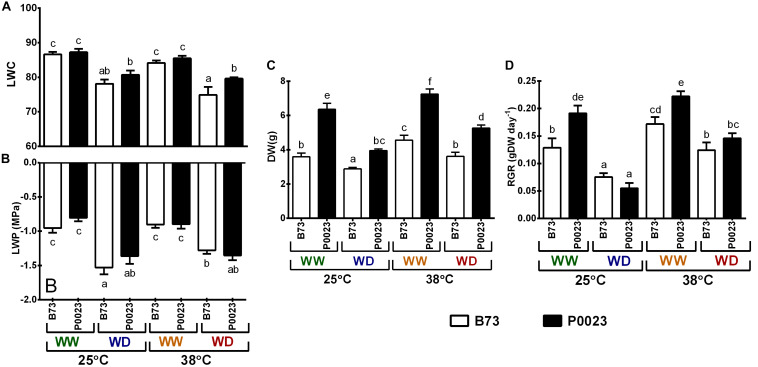
The water status and biomass of two maize genotypes (B73, P0023) grown under WW and WD conditions and acclimatized to 25 or 38°C. **(A)** Water content (LWC), **(B)** water potential (LWP) of fully expanded leaves, and **(C)** above-ground biomass of 4-week-old maize plants. **(D)** RGR during the acclimatization period. Values are mean ± SEM (*n* = 5 biological replicates). Different letters denote statistically significant differences between treatments (Duncan analysis, *p* < 0.05).

To characterize the water status of B73 and P0023 plants, LWC and LWP were estimated at the end of each experimental condition (Figures 2A,B). Well-watered (WW) plants presented LWC (Figure 2A) and LWP (Figure 2B) around or above 85% and −1 MPa, respectively, suggesting good cellular hydration. The WD conditions led to a decrease in LWC and LWP values (around or lower than 80% and lower than −1 MPa, respectively, Figures 2A,B), revealing a decrease in hydration and a considerable driving force for water uptake by the plant. Under WD38, P0023 presented a higher water content than B73, even though no significant differences were found for LWP, showing the capacity of this genotype to maintain cellular hydration under these conditions. The above-ground biomass was higher in P0023 in all the conditions and increased in both cultivars when subjected to high temperatures (WW38, Figure 2C). Under WD25, the biomass decreased in both genotypes, however, under WD38 only P0023 showed a decrease in biomass relative to WW25 (Figure 2C). To assess the biomass allocation under stress conditions, the RGR between the first day of stress and the end of the experiment were determined. The RGR was higher in P0023 in all WW conditions and decreased under WD to a similar magnitude in both genotypes.

### Effects of Drought and High Temperature on the Leaf Evaporative Cooling System

**FIGURE 3:**
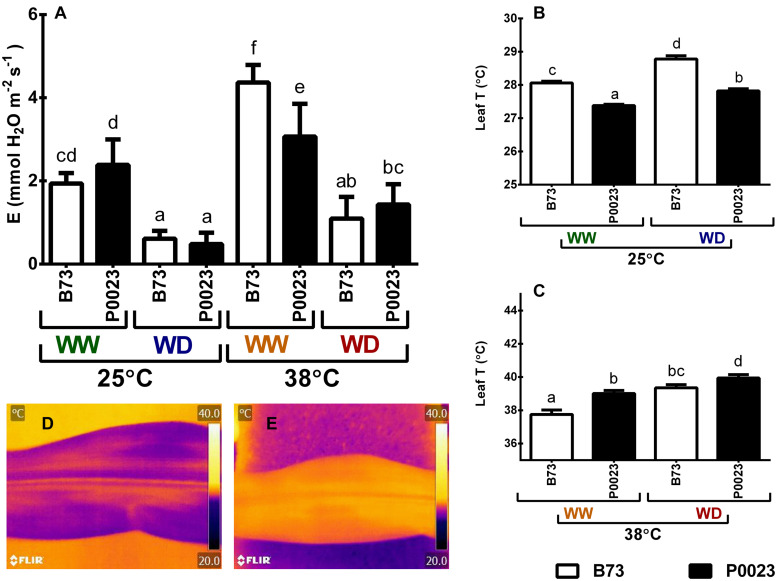
The leaf evaporative cooling system of two maize genotypes (B73, P0023) grown under WW and WD conditions and acclimatized to 25 or 38°C. **(A)** Transpiration rate, **(B)** leaf temperature at 25°C and **(C)** leaf temperature at 38°C. **(D,E)** Representative thermographic images from the leaves of 4-week-old maize plants under WW **(D)** and WD **(E)** conditions, exposed to 25°C. Values are mean ± SEM (*n* = 5 biological replicates). Different letters denote statistically significant differences between treatments (Duncan analysis, *p* < 0.05).

To understand the effect of stress conditions on the leaf evaporative cooling system, transpiration rate (E) and leaf temperature (LeafT) were measured at end of the experimental conditions. The leaf transpiration decreased significantly under WD conditions and was higher under elevated temperature (WW38, Figure 3A), in accordance with soil water availability and stomatal conductance, as assessed by leaf temperature (Figure 3B). The leaf temperature increased significantly in both genotypes under WD (Figures 3B–E) and increased in both cultivars when subjected to high temperatures (Figure 3C). Under 25°C, P0023 showed a lower LeafT than B73 in all conditions (Figure 3B), although the temperature relatively increased to 38°C for B73 (Figure 3C), in accordance with the decrease of transpiration (Figure 3A). However, the increase of leaf temperature in P0023 at 38°C did not impair the assimilation rate (Figure 1A) and ETR (Figure 1C).

### Effects of Drought and High Temperature on the CO_2_ Concentration Mechanism and Calvin–Benson–Bassham Cycle

**FIGURE 4:**
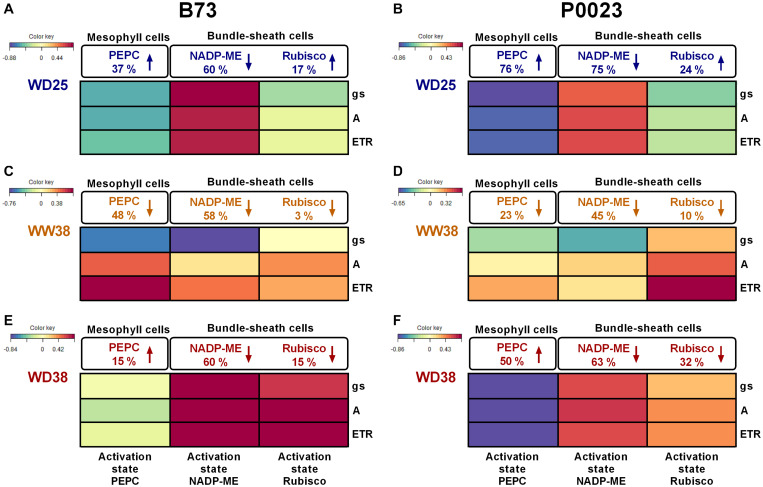
The PEPC, NADP-ME, and Rubisco activation state of two maize genotypes (B73, P0023) grown under WW and WD conditions and acclimatized to 25 or 38°C. The heatmap represents the correlation between the activation state of key photosynthetic enzymes and steady-state chlorophyll *a* fluorescence or gas-exchange parameters of two maize genotypes (B73, P0023). The canonical correlations were determined according to the effect of **(A,B)** WD at 25°C (WD25), **(C,D)** high temperatures (WW at 38°C, WW38), and **(E,F)** WD combined with high temperatures (WD38), relative to control plants (WW25). The PEPC, NADP-ME, and Rubisco activities, gs, A and ETR were measured at the respective growth temperature (25 or 38°C), in fully expanded leaves and respective extracts from 4-week-old maize plants. Differences in the catalytic activity are represented as percentage changes relative to control (WW25) in extracts measured at the same temperature (25°C for WD25 and 38°C for WW38 and WD38, Supplementary Figures 1–3). The arrow direction indicates activity increase or decrease. The different colors denote positive (red) or negative (blue) correlations between variables (*n* = 4–5 biological replicates).

To characterize the combined effects of increased temperatures and WD on CCM and CBBC, the activity of key photosynthetic enzymes was assessed, namely PEPC, NADP-ME, and Rubisco (Supplementary Figures 1–3). A multivariate canonical correlation analysis was then used to correlate enzyme activity (maximal/total activity and activation state) with photosynthetic activity and stomatal conductance (A, gs, Fv/Fm, ETR) (Figure 4 and Supplementary Figure 4).

The ratio of physiological activity to enzyme capacity was used as a proxy of the adjustment of CCM and CBBC function in response to drought and high temperature (Figure 4 and Supplementary Figures 1–3).

Under WD25, the PEPC activation state increased in both genotypes at different rates, leading to a negative correlation to the photosynthetic parameters (Figures 4A,B). While B73 showed an increase in PEPC activation state (37%, Figure 4A), by maintaining a similar Vmax (Supplementary Figure 4A) and increasing Vphysiol (Supplementary Figure 1B), P0023 increased the PEPC activation state (76%, Figure 4B) by a more pronounced decrease of Vmax (59%, Supplementary Figure 4B) relative to Vphysiol (Supplementary Figure 1B). On the other hand, both genotypes decreased the activation state of NADP-ME (Figures 4A,B) by a marked decrease of Vphysiol (Supplementary Figure 2B) and a minor decrease of Vmax (Supplementary Figures 4A,B). Regarding Rubisco, B73 and P0023 increased its activation state (Figures 4A,B) by a more marked increase of Vinitial (Supplementary Figure 3B) than Vtotal (Supplementary Figures 4A,B). Overall, B73 showed a higher positive correlation between the NADP-ME activation state (Figure 4A) and the decrease of the photosynthetic parameters (Figures 1A–C). In comparison, P0023 showed a stronger negative correlation between the activation state of PEPC (Figure 4B) and gs (Figure 1B).

Under WW38, both genotypes showed a decrease of the activation state in the carboxylation and decarboxylation enzymes (Figures 4C,D), as Vphysiol increased less than Vmax (Supplementary Figures 1, 2). Minor changes were also observed in the Rubisco activation state in both genotypes (Figures 4C,D). Strong positive correlations were observed between ETR and the activation states of PEPC in B73 (Figure 4C) and Rubisco in P0023 (Figure 4D). Moreover, a strong negative correlation was identified between gs and NADP-ME in B73 (Figure 4C).

Under WD38, a different regulation of the enzymes operating in the MCs or BSCs was observed between genotypes. The P0023 genotype increased PEPC activation state to a greater extent than B73 (50 and 15%, respectively, Figures 4E,F), with a stronger negative correlation to the decrease of photosynthetic parameters. Both genotypes decreased NADP-ME and Rubisco activation state, however, B73 showed a higher correlation to the photosynthetic parameters (Figures 4A–F).

**FIGURE 5:**
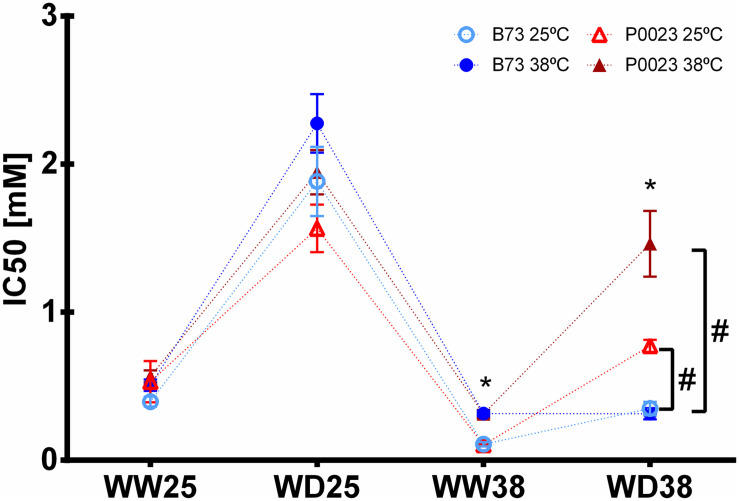
Sensitivity of PEPC activity to the inhibitor L-malate in two maize genotypes (B73, P0023) grown under WW and WD conditions and acclimatized to 25 or 38°C. The IC50 was measured at 25°C (open symbols) and 38°C (closed symbols) in extracts of fully expanded leaves from 4-week-old maize plants. Asterisks denote statistically significant differences between experimental temperature in the same genotypes and condition (*t*-test, *p* < 0.05), and hash between two genotypes in the same condition and experimental temperature (*t*-test, *p* < 0.05), *n* = 4–5 biological replicates.

The PEPC phosphorylation state was evaluated by the sensitivity of PEPC inhibition to L-malate. Overall, significantly lower sensitivity was observed in P0023 compared to B73 only in WD38, when assayed at 25 and 38°C (Figure 5). Moreover, the results showed that sensitivity to L-malate decreased with WD at 25°C (Figure 5) and was maintained at the same level of WW25 when analyzed at 38°C but increased when assayed at 25°C (Figure 5).

### Photosynthetic Performance Across Leaf in Response to Drought and High Temperature

**FIGURE 6:**
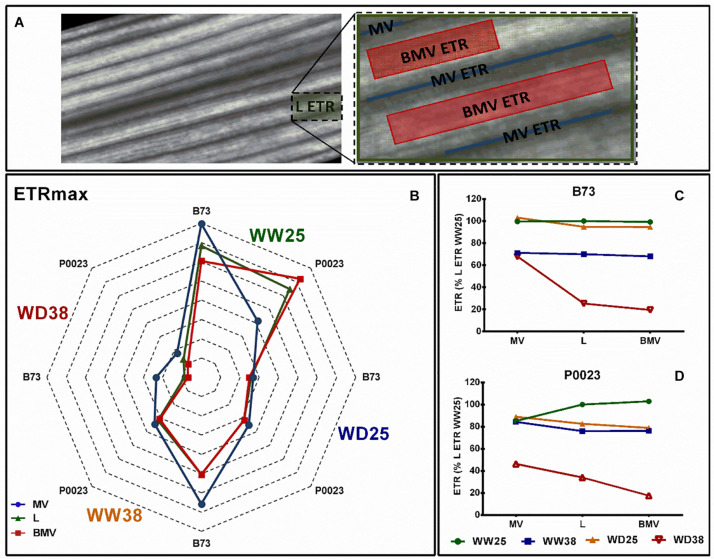
The leaf photosynthetic heterogeny of two maize genotypes (B73, P0023) grown under WW and WD conditions and acclimatized to 25 or 38°C. **(A)** Leaf (L), MVs, and BMVs AOI from a representative chlorophyll *a* fluorescence image. **(B)** Maximal ETR and **(C,D)** growth irradiance ETR extracted from AOIs in fully expanded leaves from 4-week-old maize. ETRmax estimated from a three-parameter photosynthesis-irradiance model (Platt et al., 1982), *n* = 5 biological replicates, **(C,D)** data normalized to L ETR under WW25.

Chlorophyll *a* fluorescence imaging at increasing irradiance (RLCs) was used to assess the topographic variation of the photosynthetic response across the leaf (L), MVs, and area BMVs when subjected to high temperature and drought (Figure 6A).

The ETRmax of photosynthesis varied between leaf zones, genotypes, and growth conditions (Figure 6B). In B73 at WW25, ETRmax was higher at MV ETR areas, with equal contribution between MV ETR and the inner space BMV ETR to L ETR, although in P0023 in the same conditions a higher ETR and contribution to L ETR was detected in BMV areas (Figure 6B). In WD38, both genotypes decreased the ETRmax in all zones. In WW38, a higher ETR was observed in B73 MV areas with the minor contribution to L ETR and in P0023 all the leaf zones behaved similarly (Figure 6B). Under WD38, besides ETRmax decrease in B73, the relation between the different leaf zones ETR was the same. In P0023 the difference between the three zones was less evident, but LETR showed an intermediary maximal ETR, showing a similar contribution from MVs and BMVs to total L ETR (Figure 6B).

When measured at the growth irradiance (Figures 6C,D), in B73, WD25 all the areas showed the same ETR as Leaf, but P0023 showed a slightly decrease in BMV areas (Figures 6C,D). Under stress conditions, particularly under WD38, MV areas maintained a more stable or higher ETR than the other zones. In P0023, MVs contributed more to the total L ETR than BMVs under WD38.

Additionally, under high temperature conditions, P0023 maintained a more stable quantum yield of the electron transport flux until PSI electron acceptors (OJIP data, Supplementary Figure 5A), which is highly correlated with the maintained variable fluorescence at I level measured by the chlorophyll *a* transient induction (Figure 5B).

## Discussion

### Limited Transpiration Under High Temperature/VPD as a Stress Avoidance Mechanism When Water Deficit Co-occurred With High Temperature

Two maize genotypes, B73 and P0023, were studied for their ability to withstand WD and high temperatures, in isolation or combination. Under WD at 25°C (WD25), photosynthesis was highly affected in both genotypes, reducing assimilation rate (Figure 1A), gs (Figure 1B), and ETR (Figure 1C) to the same level. Under these stress conditions, P0023 losses its capacity to grow faster, highly penalizing its RGR (Figure 2C) and biomass allocation (Figure 2D). With the data obtained, we conclude that under our experimental conditions, 7 days of WD at 25°C, P0023 did not show higher tolerance to WD, when compared to B73.

When WW plants were subjected to high temperature (WW38), both genotypes maintained near the same photosynthetic capacity (Figures 1A,C) and growth rate (Figure 2D) as control plants (WW25). However, P0023 maintained the same stomatal conductance, whereas B73 increased gs (Figure 1B). Concomitantly, the lower gs in P0023 was accompanied by a lower transpiration rate under WW38, albeit no differences were observed between genotypes under WW25 (Figure 3A). Therefore, P0023 showed low transpiration rates in response to high temperature and vapor pressure deficit (VPD), since under the WW38 experimental condition, plants were exposed to constant high VPD (2.5–4.0 kPa).

A broad genetic variation of the trait related to transpiration response to VPD conditions was extensively investigated by Sinclair and collaborators in several crops, including maize (Sinclair, 2017, 2018; Sinclair et al., 2017). Using an experimental system that allowed precise control of VPD (VPD chamber, Gholipoor et al., 2013), it was found that some maize genotypes showed early closure of stomata as VPD increases, decreasing transpiration (TRlim) and saving soil water. The trait TRlim was considered beneficial for maize production under limited water supply and therefore was genetically incorporated in the DuPont Pioneer AQUAmax hybrids (e.g., P0023, Gaffney et al., 2015). However, TRlim was found to be thermal sensitive in maize plants grown at high temperature (32°C versus 25°C, Yang et al., 2012), and in another study, some genotypes lost this characteristic when exposed for 2 days at 38°C (Shekoofa et al., 2016).

Our results demonstrated that a reduced transpiration rate was maintained in plants of P0023 acclimated to high temperature/VPD without substantial damage to carbon assimilation. Moreover, this characteristic acted beneficially as a stress avoidance mechanism when WD was associated (WD38), by maintaining high leaf hydration (Figure 2A) and photosynthetic efficiency (Figure 1).

Giuliani et al. (2005) found that maize lines with a greater concentration of roots in shallow soil layers had increased the leaf abscisic acid concentration, causing reduced stomatal conductance. Consequently, TRlim could be associated with changes in the root system, as plants with a more robust root system can explore and obtain water from deeper soil layers (Hammer et al., 2009; Adee et al., 2016). The decrease of above-ground biomass and RGR in P0023 under WD38 (Figures 2C,D) besides higher photosynthetic efficiency than B73 can also suggest that photosynthetic resources in P0023 are being used in root development.

Furthermore, aquaporins can also be associated with the regulation and expression of the TRlim trait (Choudhary et al., 2015), as several authors proposed aquaporins in the same way as key players in converting chemical signals (e.g., ABA) to hydraulic response and transmembrane CO_2_ transport (Flexas et al., 2006; Shatil-Cohen et al., 2011; Sade et al., 2014; Moshelion et al., 2015; Grondin et al., 2015; Secchi et al., 2017; Ding et al., 2020).

### Phosphoenolpyruvate Carboxylase Activation and Phosphorylation Status Contributed to the Maintenance of Photosynthesis Efficiency Under Water Deficit at High Temperature

A possible throwback of decreasing the transpiration rate under high temperature is the potential harmful increase in leaf temperature, as generally, plants use evaporative cooling to reduce it (Carmo-Silva et al., 2012; Costa et al., 2013). It is generally accepted that enzymes of CCM and Rubisco are unaffected by changes in the range of temperatures faced by plants in our experiment (Casati et al., 1997; Crafts-Brandner and Salvucci, 2002; Salvucci and Crafts-Brandner, 2004b). However, the decrease of the Rubisco activation state is usually associated with high temperatures due to an increase in catalytic misfire inhibition and decline of the regulation by heat-sensitive Rca (Salvucci and Crafts-Brandner, 2004c; Carmo-Silva and Salvucci, 2011).

Under WW38, no significant changes on the Rubisco activation state were observed (Figures 4C,D), which can be explained by the fact that in maize Rubisco is exclusively located in the chloroplast of BSC, surrounding the vascular tissue, that can offer a superior exposure to the evaporative cooling capacity, buffering the BSC temperature rise (Lundgren et al., 2014; Pignon et al., 2019). Another possible explanation is that the long-term acclimation to high temperature experienced by these plants allowed the expression of Rca isoforms more active under high temperature repairing catalytic misfire inhibition, as reported in other studies (Crafts-Brandner and Salvucci, 2002; Yin et al., 2014; Zhang et al., 2019; Kim et al., 2021).

On the other hand, the catalytic activity of carboxylating and decarboxylating enzymes was modulated by WW38 on both genotypes relative to WW25, but more extensively in B73 (Supplementary Figures 4C,D and Figures 4C,D). A possible reason for the decrease of the PEPC activation state was inhibition of physiological activity due to increased sensitivity to the inhibitor L-malate (Figure 5 and Supplementary Figure 1A), known to be mainly regulated by the PEPC kinase phosphorylation (Jiao et al., 1991; Bakrim et al., 1992; Nimmo, 2003). Nevertheless, in B73 a strong negative correlation between the PEPC maximal capacity (Vmax) and the maximum quantum yield of PSII (Fv/Fm, Supplementary Figure 4C) and a positive correlation between PEPC activation and ETR (Figure 4C) can also suggest a relation to the decline of ATP production and possible reduction of PEP regeneration by pyruvate phosphate dikinase (PPDK) in the chloroplast of the MCs, that is ATP dependent (Edwards et al., 1985; Chastain et al., 2011; Chen et al., 2014). The higher NADP-ME maximal activity (Supplementary Figures 4C,D) and lower activation state (Figures 4C,D) at high temperature mimic the changes in PEPC enzymatic capacities and respond to carbon supply and flux between MCs and BSCs (Maier et al., 2011; Wang et al., 2014). Moreover, the more considerable extent of increase of the PEPC maximal capacity than NADP-ME in both genotypes (Supplementary Figures 4C,D) can suggest that PEPC activity can be involved in the carboxylation of CO_2_ from other sources than atmospheric provenance, as the recycling of CO_2_ from BSC leakage or photorespiratory processes, usually associated with exposure to high temperatures (Hatch et al., 1995; Sage et al., 2010; Kromdijk et al., 2014).

Under WD38, when compared to WW25, both genotypes presented a decreased Rubisco activation state, due to the initial lower activity relative to total activity (Figures 4E,F and Supplementary Figure 3). This might be due to the slightly increased leaf temperature (Figure 3C), caused by the decline of gs and evaporative cooling (Figures 1B, 3), making the increase in the Rubisco catalytic activity insufficient to overcome enzyme inactivation (Crafts-Brandner and Salvucci, 2002). The substantial decrease of ETR (Figure 1C) and the consequent decline in the ADP:ATP ratio in the chloroplast might have also contributed to the observed decrease in Rubisco activation, as the repair of misfire inhibition by Rca is ATP dependent (Salvucci and Crafts-Brandner, 2004c; Carmo-Silva and Salvucci, 2011), also supported by the correlation between the Rubisco activation state and ETR in B73 (Supplementary Figure 4E). Nevertheless, P0023 increased the total activity and B73 maintained near the same as WW25 (Supplementary Figures 3, 4E,F), showing a higher physiological capacity in P0023. There was low correlation between the decrease of activation and the reduction of net photosynthetic assimilation and ETR in this genotype than B73 (Figures 1A, 5E,F).

The increase in the PEPC activation state in P0023 (Figures 4E,F) corresponds to the decrease in the sensitivity to malate (Figure 5), suggesting a different level of PEPC phosphorylation between genotypes and higher physiological functionality of PEPC in P0023. Regulation of PEPC activity by phosphorylation has been hypothesized to provide a possible link between PEPC activity and coordination of CCM upon drought and salt stress in C4 plants (Foyer et al., 1998; García-Mauriño et al., 2003; Marques da Silva and Arrabaça, 2004; Carmo-Silva et al., 2008). Increase of PEPC phosphorylation and activity under WD38 in P0023 suggest a higher CO_2_ sequestration efficiency, maintaining the carboxylation rate in MCs and the supply of C_4_ acids to BSCs adequate for Rubisco activity. Therefore, an increase in PEPC phosphorylation could be regarded as a regulatory mechanism to maintain high photosynthetic activity under WD38. Nonetheless, the differences between PEPC catalytic activity can be explained by other regulatory mechanisms, such as other post-translational modifications (Luís et al., 2016; Ruiz-Ballesta et al., 2016).

The NADP-ME maximal activity was reduced by half, relative to WW38, demonstrating the negative effect of drought at 25°C (Supplementary Figures 4A,B) and 38°C (Supplementary Figures 4C,D relative to Figures 4E,F). Several authors reported the decrease of NADP-ME activity under WD (Du et al., 1996; Marques da Silva and Arrabaça, 2004; Carmo-Silva et al., 2008), but to the best of our knowledge, no changes in NADP-ME activity have been previously reported in maize plants acclimatized to high temperature and subjected to WD. Nevertheless, the plasticity of the decarboxylating process in maize plants under stress conditions was identified by other authors (Chapman and Hatch, 1981; Wingler et al., 1999; Bellasio and Griffiths, 2014), and alternative decarboxylating processes, as the synthesis of aspartate as a major translocated C_4_ acid, can compensate the decrease of NADP-ME decarboxylating activity under our experimental conditions (Furbank, 2011; von Caemmerer and Furbank, 2016).

### Electron Transport Rate Stability Near Vascular Tissues Contributed to the Supply of Chemical Energy for an Effective CO_2_ Concentrating Mechanism Under Water Deficit at High Temperature

The P0023 genotype showed a higher maximum quantum yield of PSII (Figure 1D) and stable quantum yield of the electron transport flux until the PSI electron acceptors (Supplementary Figure 5A) under high temperatures and a higher contribution of MV ETR to whole L ETR under WD38 (Figure 6C). These results demonstrated the higher efficiency in producing chemical energy (ATP and reduction power) in this genotype under higher temperature. The maize MVs are anatomically characterized by highly differentiated BSCs surrounded by MCs, forming concentric circles around the vasculature (Lundgren et al., 2014). Adenosine triphosphate and reduction power are cofactors of most photosynthetic enzymes, and reduction power is moved from MCs to BSCs through malate decarboxylation (Furbank, 2011) and the shuttle of 3-PGA and triose phosphate (Hatch, 1987; Bräutigam and Weber, 2010). Thus, the observed superior ETR stability in these zones (MVs) and the maintenance of the physical integrity components of the photosynthetic apparatus in MCs and BSCs can be regarded as crucial for successful acclimation of photosynthesis to high temperature conditions. Moreover, reduction of stomatal conductance under high temperature as a water-saving mechanism in P0023 and maintenance of hydraulic conductance can also act preventing desiccation and maintenance of highly productive MCs and BSCs surrounding the vascular tissues (Figures 1B, 6D; Sunita et al., 2014; Sade et al., 2014; Moshelion et al., 2015).

## Conclusion

In summary, the limited transpiration rate under high temperature/VPD, together with higher efficiency in the regulation of CCM contributed to the maintenance of a better physiological status in P0023 under high temperature and/or extended drought. These characteristics can allow water conservation in initial periods of soil drying, without substantial crop production damage. Genotypes with the same traits may be suitable for crop production in environments with high temperature that experience regular water shortage periods. Furthermore, high throughput screening of maize hybrids under similar experimental settings, but mimicking field light conditions, can select genotypes with the same characteristics and potential for more stable production in warmer and drier conditions, helping to overcome future throwbacks in food production.

## Data Availability Statement

The original contributions presented in the study are included in the article/Supplementary Material, further inquiries can be directed to the corresponding author/s.

## Author Contributions

PC planned and carried out the experiments, analyzed, interpreted the results, and took the lead in writing the manuscript. EC-S and JS contributed to the interpretation of the results and supervised the research. AS and MV provided critical feedback. All authors discussed the results and contributed to the final manuscript.

## Conflict of Interest

The authors declare that the research was conducted in the absence of any commercial or financial relationships that could be construed as a potential conflict of interest.

## Publisher’s Note

All claims expressed in this article are solely those of the authors and do not necessarily represent those of their affiliated organizations, or those of the publisher, the editors and the reviewers. Any product that may be evaluated in this article, or claim that may be made by its manufacturer, is not guaranteed or endorsed by the publisher.
