# Supplementary material for: Efficient Regulation of CO2 Assimilation Enables Greater Resilience to High Temperature and Drought in Maize
Source: Front Plant Sci. 2021 Jul 26;12:675546. doi: 10.3389/fpls.2021.675546 (PMC8350398; doi:10.3389/fpls.2021.675546)
Supplement: Supplementary file 1 [file Data_Sheet_1.docx]

Supplementary Material

Supplementary Figures





**Supplementary Figure 1.** Effect of high temperature and drought on PEPC activity (expressed by total soluble protein, TSP) and activation state in two maize genotypes, B73 and P0023. **(A–C)** Maximal (Vmax) and physiological (Vphysiol) activities and activation state were measured at 25°C and **(D–F)** at 38°C in extracts of fully expanded leaves from 4‐week‐old maize plants under well‐watered (WW) and water deficit (WD) conditions and exposed to control (25°C) and heat stress conditions (38°C). **(A and D)** Vmax, **(B and E)** Vphysiol, **(C and F)** activation state. Values are means ± sd (n = 4–5 biological replicates). Different letters denote statistically significant differences between treatments (Duncan analysis, p < 0.05)





**Supplementary Figure 2.** Effect of high temperature and drought on NADP-ME activity (expressed by total soluble protein, TSP) and activation state in two maize genotypes, B73 and P0023. **(A–C)** Maximal (Vmax) and physiological (Vphysiol) activities and activation state were measured at 25°C and **(D–F)** at 38°C in extracts of fully expanded leaves from 4‐week‐old maize plants under well‐watered (WW) and water deficit (WD) conditions and exposed to control (25°C) and heat stress conditions (38°C). **(A and D)** Vmax), **(B and E)** Vphysiol, **(C and F)** activation state. Values are means ± sd (n = 4–5 biological replicates). Different letters denote statistically significant differences between treatments (Duncan analysis, p < 0.05)





**Supplementary Figure 3.** Effect of high temperature and drought on Rubisco activity (expressed by total soluble protein, TSP) and activation state in two maize genotypes, B73 and P0023. **(A–C)** Rubisco initial (Vi) and total (Vt) activities and activation state were measured at 25°C and **(D–F)** at 38°C in extracts of fully expanded leaves from 4‐week‐old maize plants under well‐watered (WW) and water deficit (WD) conditions and exposed to control (25°C) and heat stress conditions (38°C). **(A and D)** Total activity (vi), **(B and E)** initial activity (vt), **(C and F)** activation state. Values are means ± sd (n = 4–5 biological replicates). Different letters denote statistically significant differences between treatments (Duncan analysis, p < 0.05)


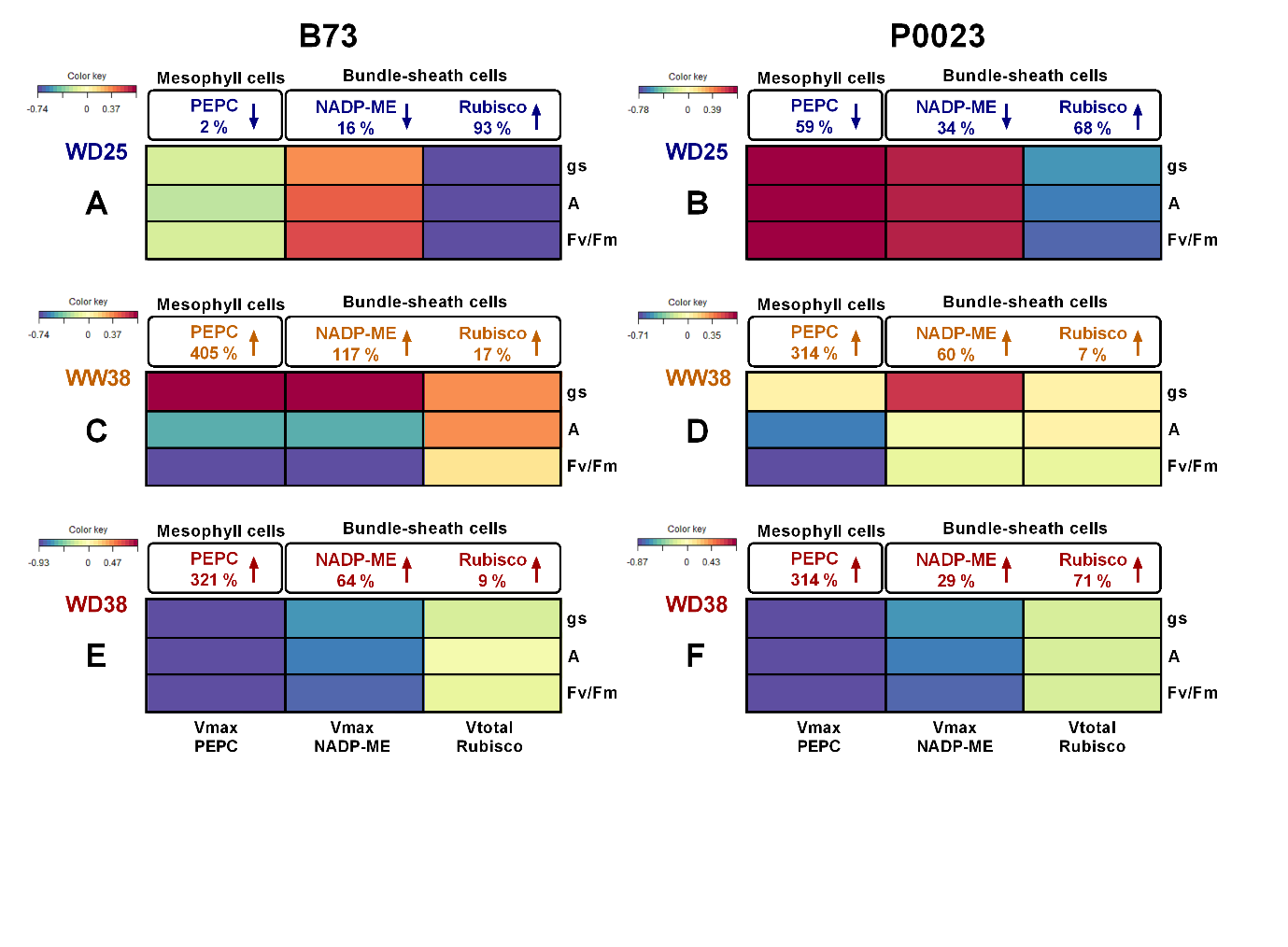


**Supplementary Figure 4 –** PEPC, NADP-ME and Rubisco maximal capacity of two maize genotypes (B73, P0023) grown under well-watered (WW) and water deficit (WD) conditions and acclimatized to 25ºC or 38ºC. Heatmap represents the correlation between maximal/total activity (Vmax/Vtotal) of key photosynthetic enzymes and steady-state chlorophyll a fluorescence or gas-exchange parameters of two maize genotypes (B73, P0023). Canonical correlations were determined according to the effect of (**A-B**) water deficit at 25 ºC (WD25), (**C-D**) high temperatures (well-watered at 38ºC, WW38) and (**E-F**) water deficit combined with high temperatures (WD38) relative to control plants (WW25). Vmax PEPC, Vmax NADP-ME (Vmax), Total Rubisco activity (Vtotal), gs, A and ETR were measured at the respective growth temperature (25ºC or38 ºC), in extracts or fully expanded leaves of 4-week-old. Differences in the catalytic activity are represented as percentages changes relative to control (WW25) in extracts measured at the same temperature (25ºC for WD25 and 38 ºC for WW38 and WD38). Arrows direction indicate activity increase or decrease. Different colours denote positive (red) or negative (blue) correlations between variables (n = 4-5 biological replicates).


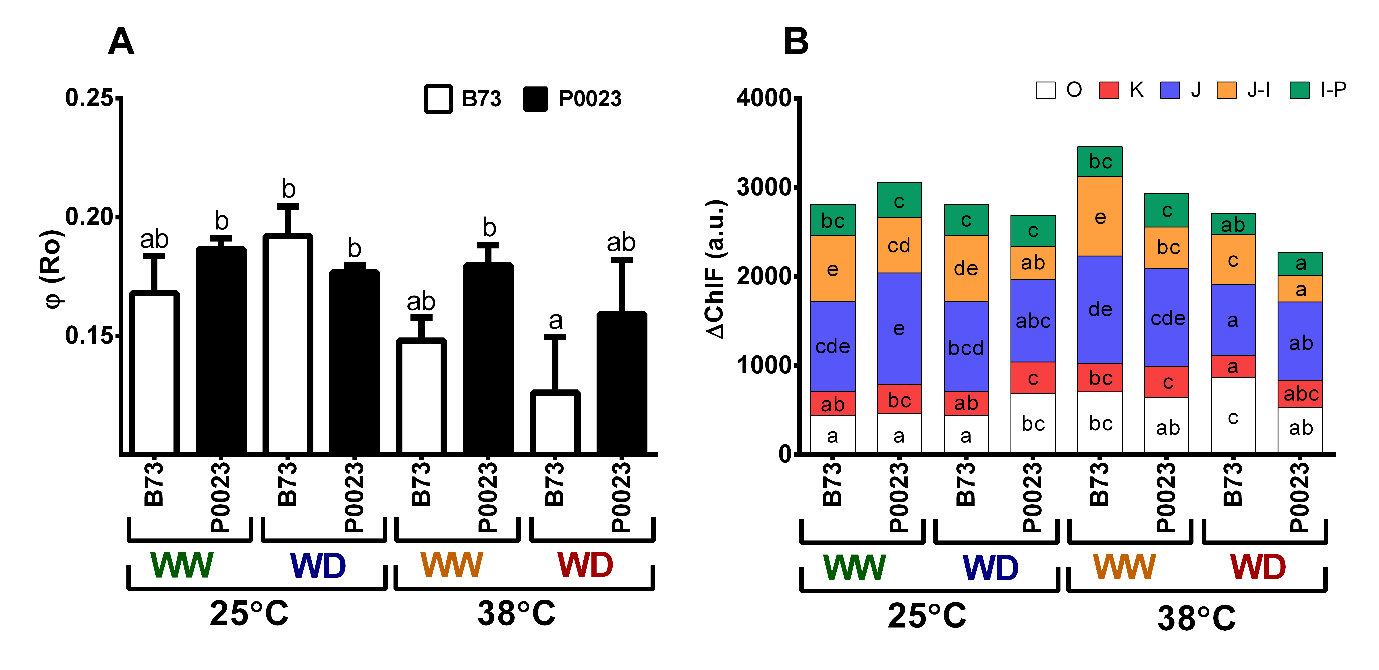


**Supplementary Figure 5**- Chlorophyll *a* fluorescence induction in two maize genotypes (B73, P0023) grown under well-watered (WW) and water deficit (WD) conditions and acclimatized to 25ºC or 38ºC. **(A)** Quantum yield of the electron transport flux until the PSI electron acceptors and **(B)** the amplitude of variable fluorescence of chlorophyll *a* transient fluorescence rise (OKJIP) were measured in dark-adapted fully expanded leaves of maize 4-week-old plants. Different coloured bars indicate the minimal fluorescence O, (Fo, white) and changes in the amplitude of variable fluorescence intensities (ΔChlF), K (F300 µs- Fo, red), J (F2ms- Fo, blue), J-I (F30ms- F2ms, orange) and I- P (FFm- F30ms, Fm=maximal fluorescence, green). Values are means ± SEM (n = 5 biological replicates). Different letters denote statistically significant differences between treatments (Duncan analysis, p < 0.05).
